# Limb salvage of an infant with infantile fibrosarcoma using TRK inhibitor larotrectinib

**DOI:** 10.3332/ecancer.2023.1575

**Published:** 2023-07-19

**Authors:** Shubham Sahni, Sameer Rastogi, Richa Yadav, Adarsh Barwad

**Affiliations:** 1 Department of Medicine, All India Institute of Medical Sciences, New Delhi 110029, India; 2 Department of Medical Oncology, All India Institute of Medical Sciences, New Delhi 110029, India; 3 Department of Radio-diagnosis and Interventional Radiology, All India Institute of Medical Sciences, New Delhi 110029, India; 4 Department of Pathology, All India Institute of Medical Sciences, New Delhi 110029, India

**Keywords:** infantile fibrosarcoma, larotrectinib, NTRK3-ETV6

## Abstract

Infantile fibrosarcoma (IFS) is an extremely rare locally aggressive soft tissue tumour of childhood. Primary therapy involves complete surgical resection with or without chemotherapy. However complete surgical resection might not be feasible in all cases and so requires other modalities for further management. We report the case of a male infant from Bangladesh with a locally advanced IFS of the leg which was partially resected. The patient received adjuvant chemotherapy which was complicated by the development of chemotherapy-related veno-occlusive disease and had to be discontinued. Thereafter he was referred to our dedicated sarcoma oncology clinic in India for further management. The parents of the child refused amputation of the limb. The tumour tested positive for NTRK3-ETV6 gene fusion and after discussion in multidisciplinary clinic, targeted therapy using oral NTRK inhibitor larotrectinib was started. The patient had complete response at the end of 8 months of treatment with larotrectinib. This is the first report from the Indian subcontinent and we encourage that these children should be referred to specialist clinics for appropriate multidisciplinary management for best outcomes.

## Introduction

Infantile fibrosarcoma (IFS) is a locally aggressive soft tissue tumour of childhood with low propensity for distant metastasis. More than 75% of IFS are reported within 1 year of life [1]. IFS demonstrates a wide histopathologic spectrum with sheets or bundles of spindle-shaped cells and may occasionally display sheets of immature polygonal or round cells with frequent mitoses [2]. Immunohistochemistry (IHC) in IFS reveals positivity for vimentin, desmin, smooth muscle actin and muscle-specific actin [3]. Surgical resection forms the mainstay of management for resectable tumours. Chemotherapy can be used in the neoadjuvant setting to allow safe resection in cases where satisfactory upfront resection is not possible [4]. The utility of adjuvant chemotherapy or radiotherapy remains controversial and is not routinely recommended [5, 6]. Conventional chemotherapeutic options for IFS include vincristine + actinomycin-D (VA) or vincristine + actinomycin – D + cyclophosphamide (VAC) based regimens [6] with variable response rates and toxicity profiles.

Several cytogenetic abnormalities underlie an IFS, the most consistent of which include translocation t(12;15) (p13;q25) producing ETV6-NTRK3 gene fusion [7]. The NTRK belongs to a family of transmembrane tyrosine kinases. Cytogenetic aberrations such as chromosomal translocations or deletions can produce fusion of the C-terminal NTRK domain with an N-terminal of a separate gene [8]. These fusions lead to the formation of a chimeric oncoprotein characterised by ligand-independent receptor dimerisation and phosphorylation. The result is constitutive TRK receptor activation and this may play a role in oncogenesis [9, 10]. The presence of NTRK fusion offers a targeted therapeutic choice for patients with IFS and has the potential to avoid the potentially toxic effects of conventional chemotherapy.

Larotrectinib is a highly selective and potent inhibitor of TRK kinase and has been shown to have good response rates in TRK fusion cancers [11–13]. Larotrectinib has been approved for patients with solid tumours harbouring NTRK fusion that are either locally advanced, have metastatic disease, or cannot be completely resected without significant loss of function [14]. However, data from low and middle-income countries (LMIC) for the use of larorectinib in IFS is not available because of frequent misdiagnosis, lack of readily available molecular markers at peripheral hospitals and poor accessibility of larotrectinib. We hereby describe a child with IFS who had a complete response to this drug after 8 months of treatment and limb salvage was achieved.

## Case

**Figure 1.**
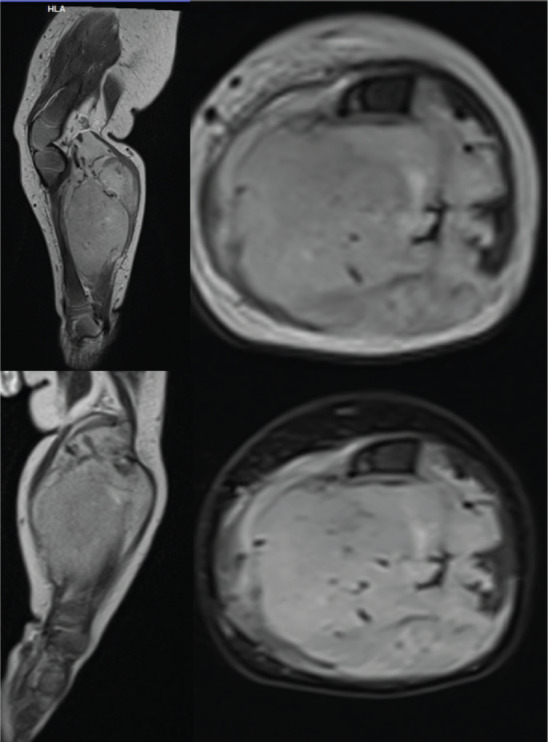
Pre-operative MRI images of the left leg showing a large lobulated invasive soft tissue mass lesion of 3.5 × 5 × 6 cm size in the upper and mid-leg involving postero-medial compartment with anterior displacement of the tibia with its bowing without any abnormal marrow signal change. Mass is hypointense on T1WI and heterogeneously hyperintense on T2WI. Mass is mainly involving the muscular structures with evidence of neuro-vascular encasement.

An 8-month-old boy, a resident of Bangladesh presented with a progressively enlarging swelling in the left leg which was present since birth. There were no associated neurovascular deficits or constitutional symptoms. Magnetic resonance imaging (MRI) of the left leg revealed a large, lobulated and invasive soft tissue mass of 3.5 × 5 × 6 cm size in the posteromedial compartment of the left leg. The mass was encasing the neurovascular bundle of the left leg (Figure 1). A wide local excision was attempted in the patient’s native country. However, it was an incomplete resection given the involvement of the neurovascular bundle.

**Figure 2.**
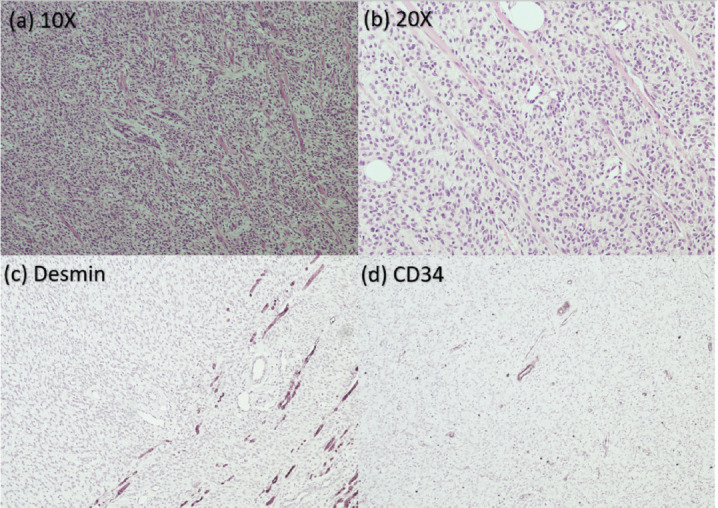
Pathological images from the resected tumour specimen. (a): Haematoxylin-eosin staining at 10× magnification. (b): Haematoxylin-eosin staining at 20× magnification. (c): Negative IHC for desmin. (d): Negative IHC for CD34.

**Figure 3.**
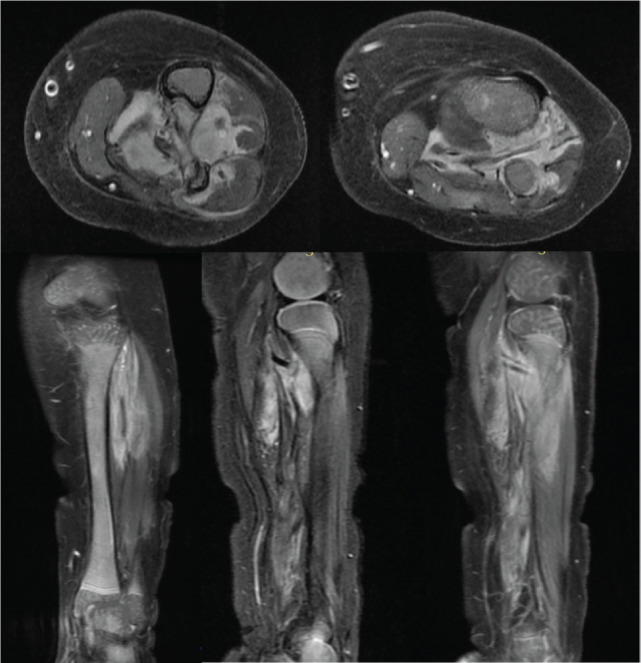
MRI scan images of the left leg showing local recurrence after wide local resection and 17 cycles of VAC-based chemotherapy.

Histopathology and IHC was consistent with the diagnosis of IFS (Figure 2). Chemotherapy with VAC regimen (Vincristine at 0.05 mg/kg + Actinomycin-D at 50 mcg/kg + Cyclophosphamide at 50 mg/kg) was given in view of incomplete resection. It was complicated by the development of chemotherapy-related veno-occlusive disease (VOD). A review of the drug charts revealed that the child had received a higher-for-age dose of Actinomycin-D. Thereafter, he was presented to our sarcoma oncology centre in India for a decision regarding further management. A repeat MRI scan of the left leg was suggestive of a residual tumour (Figure 3). This case posed a dilemma to us since the residual tumour tissue warranted revision surgery which could have culminated in limb amputation for an 8-month-old child. The second option was chemotherapy which had already been tried for the child with potentially life-threatening complications.

**Figure 4.**
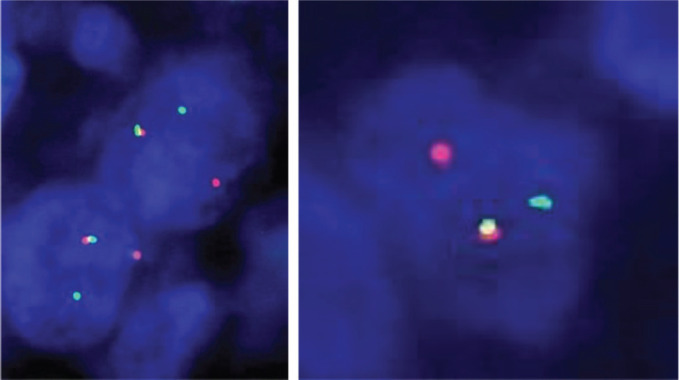
FISH on interphase cell showed evidence of ETV6 translocation/ rearrangement {ETV6-NTRK3: t(12;15) (p13;q25)}.

**Figure 5.**
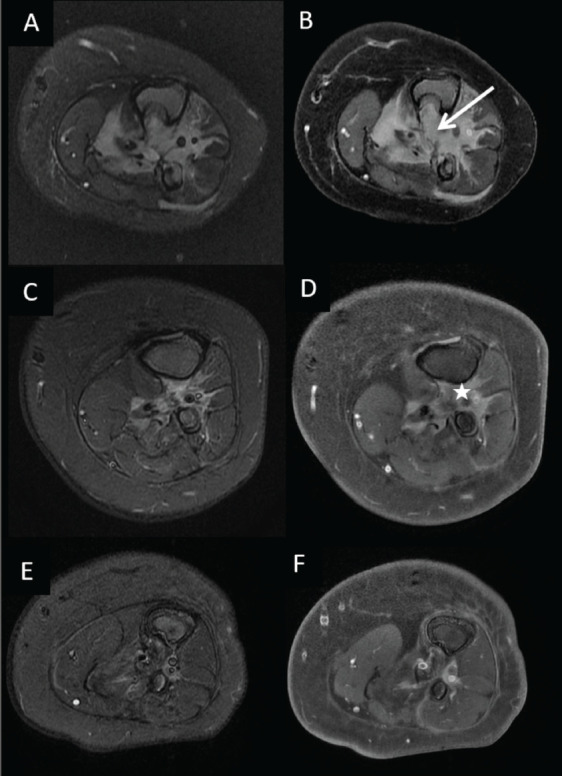
Pre-chemotherapy; (a): axial T2W (fat saturated) and (b): contrast-enhanced T1W (fat-saturated) MRI images showing Ill-defined homogenously enhancing mass lesion in the Interosseous space (arrow) causing erosion of tibia and fibula. (c, d): Corresponding post-chemotherapy MR images showing significant reduction of the mass lesion with minimal enhancing residual soft tissue lesion at the end of 3 months of larotrectinib (Asterix) and (e, f) complete response after 8 months of therapy.

A histopathology review and fluorescent-*in-situ* hybridisation (FISH) were done on the tumour sample which showed the evidence of ETV6-NTRK3: t(12;15) (p13;q25) translocation/rearrangement (Figure 4). Next-generation sequencing (NGS) performed on the formalin-fixed paraffin-embedded block revealed fusion of ETV6:NTRK3 (5’-chr12:12022903:+ and 3’-chr15:88483984:-). This offered us the opportunity to use targeted therapy in the form of oral NTRK inhibitor Larotrectinib and avoid potentially mutilating surgery or toxic chemotherapeutic regimens. Larotrectinib oral suspension was procured and administered to the child at a dose of 100 mg/m^2^. Interim imaging for response assessment done after 3 months of treatment with larotrectinib demonstrated a dramatic reduction in the size of the tumour with only minimal residual enhancing soft tissue lesion. The therapy was continued for 8 months and repeat imaging was suggestive of a radiologic complete response (Figure 5). The child is currently doing well and is continuing on oral larotrectinib suspension without any immediate or delayed toxicities.

## Discussion

This report highlights the utility of oral NTRK inhibitor larotrectinib in locally advanced IFS and the challenges faced in the management of the same in LMIC settings. IFS are rare paediatric tumours with the median age of presentation being 1.43 months [15] and are more common among boys compared to girls [15]. Our patient was a boy and had symptom onset at birth. The most common sites of involvement are the distal extremities [16] as in our case. Rare sites of involvement reported in the literature are the chest wall, retroperitoneum, axilla, tongue, lung and heart [17–19].

The mainstay of conventional management of IFS involves primary surgery for complete resection of the tumour and at the same time aiming for maximum functional and anatomic preservation. However, complete conservative resection is seldom feasible as was demonstrated in a retrospective case review of 56 infants by Orbach *et al* [6] with only 21% of the infants achieving complete resection. This was described in multiple reviews and a complete microscopic tumour eradication is seldom feasible with R0 resection rates varying from 48%–51% [15]. There is no role of adjuvant chemotherapy after R0 or R1 resection of the primary tumour at present [15]. Chemotherapy with conventional agents is recommended for incompletely resected or unresectable tumours which can later be taken up for delayed surgery [5, 15]. In a review by the European Paediatric Soft Tissue Sarcoma Study Group (EpSSG), 25 children with inoperable or incompletely resected IFS were administered with VA chemotherapy with an overall response rate of 68% [15]. Seven of these had grades III–IV toxicity including three children with VOD and one child succumbing to an overdose due to medication error. In the present case too, VAC chemotherapy was administered for residual tumour post-resection. The child received the full dose for actinomycin-D rather than the recommended 50% dose reduction and developed VOD. Chemotherapy administration in young infants is also a challenging affair considering the need for venous access, accurate dose calculation and the risk of systemic infection. Weekly hospital visits for chemotherapy are an additional burden for the caregiver and place the infant at an increased risk of hospital-acquired infections. Although radiotherapy is an option for local control after inadequate resection, especially in IFS of the chest or retroperitoneum, the use of radiotherapy for limb IFS (as in our case) has the potential risk of hampering growth and growth plate toxicity especially in young children. This was one of the reasons for not utilising radiotherapy in this infant. Also, targeted therapy offers significant clinical responses which have not been reported by radiotherapy alone.

NTRK inhibitors have changed the treatment landscape for IFS. Larotrectinib is an oral, highly selective and potent inhibitor of TRK kinase and has been shown to have good response rates in TRK fusion cancers like IFS [11]. Demonstration of ETV6-NTRK3 gene fusion can be done using FISH, reverse transcriptase polymerase chain reaction or genomic sequencing [20]. As in this case, we used FISH and NGS to detect the gene fusion. ETV6-NTRK3 gene fusion is among the most consistent molecular abnormality seen in IFS and was detected in 74% and 87% of IFS patients in reviews by Cooperative Weichteilsarkom Studiengruppe (CWS) [21] and EpSSG [15], respectively. However, the patient hailed from a different country and was not offered genetic testing for the NTRK fusion product at the index presentation over there. There is a lack of awareness regarding these novel therapeutic options and also limited availability of genetic testing for these tumours. Ours is a dedicated sarcoma oncology clinic and we utilised genetic testing for NTRK fusion followed by administration of larotrectinib in this case. This was followed by complete response of the tumour to treatment and offered the opportunity for limb salvage by avoiding a potential amputation and also obliviated the need for further doses of toxic chemotherapy. Since it’s an orally administered agent, there was no need for multiple day-care visits or any invasive lines for chemotherapy administration. Larotrectinib is associated with rare adverse events including raised alanine aminotransferase, raised aspartate aminotransferase, nausea and neutropenia [22]. In our case, the agent was well-tolerated without any reported adverse events.

Data concerning the use of larotrectinib for children with IFS have demonstrated overall response rates above 90% [14]. Neoadjuvant use of larotrectinib was also explored in a study by DuBois *et al* [23] on five patients with localised IFS followed by surgery. Three of these achieved complete or near-complete pathologic response and larotrectinib could be discontinued for them post-surgery. Neoadjuvant use of NTRK inhibitors would have multiple merits including finite treatment rather than an indefinite treatment in the relapsed/refractory setting.

A handful of similar reports utilising NTRK inhibitors in the treatment of IFS have been reported recently, but these are mainly from high-income countries (HIC) [24–26]. Ours is the first-ever reported case of successful treatment of IFS using Larotrectinib from any LMIC to the best of our knowledge. The rarity of IFS and the unawareness about the utility of targeted therapy against NTRK fusion in these tumours are some of the prime reasons for the limited use of these agents. Other issues include the high cost of treatment and limited availability of larotrectinib, especially in resource-limited nations. The patient described above is being continued on larotrectinib. Larotrectinib should be considered for refractory, recurrent or metastatic tumours [14]. There are no current recommendations on the duration of treatment with larotrectinib in this setting.

There are multiple issues with the use of these novel targeted agents in LMIC. Use of these agents requires advanced molecular testing which is often an expensive affair. In addition, there are issues pertaining to difficult access and potential shortage as most of the targeted agents are either unavailable in LMICs or have to be procured from the HIC. This poses logistic issues and increases the financial stress to an over-burdened health care system. Most targeted agents are very expensive and it adds to the financial toxicity of the patients. All this requires the use of LMIC-adapted strategies to cope with these issues. Prompt referral to dedicated sarcoma oncology clinics and improving the awareness about molecular testing for rare tumours like IFS using FISH can significantly improve outcomes in LMIC. Exploring the role neoadjuvant NTRK inhibitors followed by surgical resection after a satisfactory response would provide a finite treatment duration rather than for prolonged or indefinite duration in the adjuvant setting. This would significantly tackle the shortage and financial toxicity related to its use. The use of novel oral agents like larotrectinib in LMIC also reduces the number of hospital visits and the costs associated with administration of intravenous chemotherapy.

There are some issues that need to be addressed. First, there is no robust data on the duration of treatment and/or the risk of relapse on discontinuation of larotrectinib. In our case, we plan on continuing the agent till progression. It would not be an issue since it’s an oral and well-tolerated drug. Also, there is no evidence of outcomes on discontinuation or choice of salvage therapies in the case of a relapse. Cost is an issue but the drug is being made available to the patient through conditional access from an indigenous brand. As data build up on this entity, we might decide to stop/taper the drug. Secondly, data pertaining to long-term outcomes of patients of IFS on larotrectinib therapy are limited. Most of the reports are individual patient reports reporting finite time point response rate assessment, making it difficult to report long-term prognosis and overall survival. Nevertheless, with increasing recognition of the utility of this drug in IFS, long-term data with clinically relevant end points might become available in years to come. In a pooled analysis of NTRK fusion cancers by Hong *et al* [22], 29 patients with IFS were included and the median duration of response was ‘not estimable’ in this population at the time of analysis and 27 out of 28 patients responded with response rates of 96%. However, in this pooled analysis, multiple other histologies harbouring NTRK fusion were also taken and the median progression-free survival (PFS) was 28.3 months with 67% of patients being progression-free at 12 months. In the entire cohort, the estimated proportion of patients surviving at 12 months was 88% (mOS = 44.4 months). The rates for IFS subset were not estimable at the time of analysis.

In a nutshell, after addition of larotrectinib to the armamentarium for the treatment of IFS, the scenario has changed and it is prudent that more clinical resources, trials and expert teams be made available in LMIC to add to the existing evidence which can help improve the outcome of this rare tumour.

## Conclusion

In this era of targeted therapy for cancers, NTRK inhibitors could prove to be a useful addition to the therapeutic armamentarium for IFS and there is a need for LMIC-adapted strategies for the use of targeted agents for best outcomes.

## Conflicts of interest

No conflicts of interest.

## Funding

We did not receive any funding for this manuscript and have no financial interests.
